# Immunonutrition in patients with cyctic fibrosis leads to drop of serum amyloid A and increase of oxidative stress

**DOI:** 10.3164/jcbn.16-83

**Published:** 2017-02-24

**Authors:** Ondrej Hloch, Jiri Charvat, Libor Fila, Havlin Jan

**Affiliations:** 1 Department of Internal Medicine, 2nd Faculty Medicine of Charles University and Faculty Hospital Prague Motol, Prague, V Úvalu 84, 150 06 Prague 5, Czech Republic; 2 Department of Pneumology, 2nd Faculty Medicine of Charles University and Faculty Hospital Prague Motol, Prague, V Úvalu 84, 150 06 Prague 5, Czech Republic; 3 3rd Department of Surgery, 1st Faculty Medicine of Charles University and Faculty Hospital Prague Motol, Czech Republic

**Keywords:** immunonutrition, oxidative stress, inflammatory parameters, cystic fibrosis, malnutrition

## Abstract

The aim of the present study is to evaluate of the impact of immunonutrition on parameters of oxidative stress and inflammation in patients with cystic fibrosis and malnutrition. In the 30 patients with cystic fibrosis and long-term enteral nutrition support for malnutrition the effect of standard and immunonutrion sipping on oxidative stress and inflammatory activity parameters was compared. Malonyldialdehyde (MDA) as parameter of oxidative stress and serum amyloid A (SAA), interleukin 1 and 6, hsCRP, IgM, IgA, IgG as parameters of inflammatory activity were examined. Immunonutrition decreased SAA to 17.6 mg/L comparing to 25.6 mg/L when standard nutrition was given (*p* = 0.014). MDA was 0.66 µM on standard and 0.96 µM on immunonutrition support (*p*<0.01). The significant negative correlation was recorded between MDA and SAA, hs-CRP, interleukin 6, IgA and IgG. In conclusion, the application of immunonutrition in patients with cystic fibrosis and malnutrition is associated with drop of SAA but with the rise of MDA.

## Introduction

Cystic fibrosis is associated with the serious dysfunction of the respiratory and gastrointestinal systems. Its treatment should prevent not only deterioration of the respiratory function but also manifestation of malnutrition.^(1)^ The significant part of the patients suffering from cystic fibrosis is supported with arteficial nutrition because malnutrition negatively influences the prognosis and is associated with the frequent complications.^(1)^ Nutritional support is most often applied as enteral nutrition.

Nutrition is important not only for the appropriate energetic and protein balance but it may modify parameters of inflammation and oxidative stress as well. The impact of arginine, glutamine, omega-3 fatty acids and nucleotides on the parameters of inflammatory activity and oxidative stress might be beneficial. It was demonstrated in the patients during surgical procedure or with oncology disease.^(2–4)^ However the application of immunonutrition given as enteral sipping on the parameters of oxidative stress and inflammation has not yet been examined in the patients with cystic fibrosis. It is a reason why in our study the effect of the immunonutrition preparation Impact (Nestlé Co., Vevey, Switzerland) on oxidative stress and selected parameters of the inflammation is compared with the routine polymeric sipping Nutridrink (Nutricia Co., Amsterdam, Netherland).

## Patients and Methods

The study was realized during years 2014–2015 in Department of Internal Medicine of Faculty Hospital Prague Motol, Czech Republic. The patients with diagnosis of cystic fibrosis followed in the Department of Pneumology who reached 18 years and received enteral nutrition support by sipping for one year at least were included into study after they signed the written informed consent approved by local ethical committee. The protocol complied with principles in the Declaration of Helsinki.

Nutridrink (Nutricia Co.) had been used as a nutrition support applied by sipping. The patients with good nutritional status without need of sipping support or who received enteral nutrition by any tubing system or parenteral nutrition were excluded. The majority of patients received also substitution of pancreatic enzymes.

Before entering into the study the presence of acute infection or acute exacerbation of chronic infection was ruled out by careful clinical examination including lung function testing.

Malonyldialdehyde (MDA) as a parameter of oxidative stress and hsCRP (high sensitive C-reactive protein), SAA (serum amyloid A), interleukin (IL) 1 and 6, antibodies IgM, IgA and IgG as parameters of inflammatory activity were examined. MDA was examined using spectrophotometric method (Agilent Cary 60, Melbourne, Australia). SAA was determined with immunefelometric method (Immage 800, Canton, MA), hsCRP and immunoglobulins with immunoturbidemetry (analyzator Advia 1800, Munich, Germany). IL1 and IL 6 were examined with ELISA method (Affymetrix, eBioscience, San Diego, CA).

After clinical, inflammatory and oxidative stress parameters were examined the patient switched from Nutridrink to immunonutrition (Impact Nestle,Vevey, Switzerland) that was given in corresponding energy amount for the period of 8 weeks. After 8 weeks, the examination of the followed parameters was repeated and the patients returned to Nutridrink. After another 8 weeks, the examination was repeated and evaluated once more.

Comparison of composition of Nutridrink and Impact—energy intake of 100 kcal is summarized in Table 1.

### Statistic evaluation

Numerical values are expressed as mean ± SD. Normality of the distribution of data was assessed by visual inspection of histogram and normal probability plot. Parametric continuous data were compared using Student *t* tests and non-parametric using Mann-Whitney tests. Pearson or Spearman’s bivariate correlation analysis was used to examine the relationship between parametric and non-parametric numerical variables, respectively. All statistical analyses were performed using Statistica 12.0 (StatSoft, Tulsa, OK).

## Results

Thirty patients participated in the study, 22 men and 8 women. The average age was 24.7 ± 3.6 years. 13 patients suffered from diabetes mellitus, 27 received the substitution with pancreatic enzymes and 14 were treated for metabolic bone disease. Energy intake given by sipping was 557 ± 108 kcal daily.

The changes of body weight, systolic and diastolic blood pressure, heart rate and BMI during the study are summarized in Table 2. Systolic and diastolic blood pressure as well as heart rate significantly dropped down after 8 weeks of Impact application but they returned after Nutridrink was restarted.

The changes of MDA concentration and the inflammatory parameters are shown in Table 3. The significant elevation of MDA and drop of SAA were recorded after 8 weeks of Impact application. However, when Nutridrink was restarted MDA went down and SAA has again increased.

**Fig. 1:**
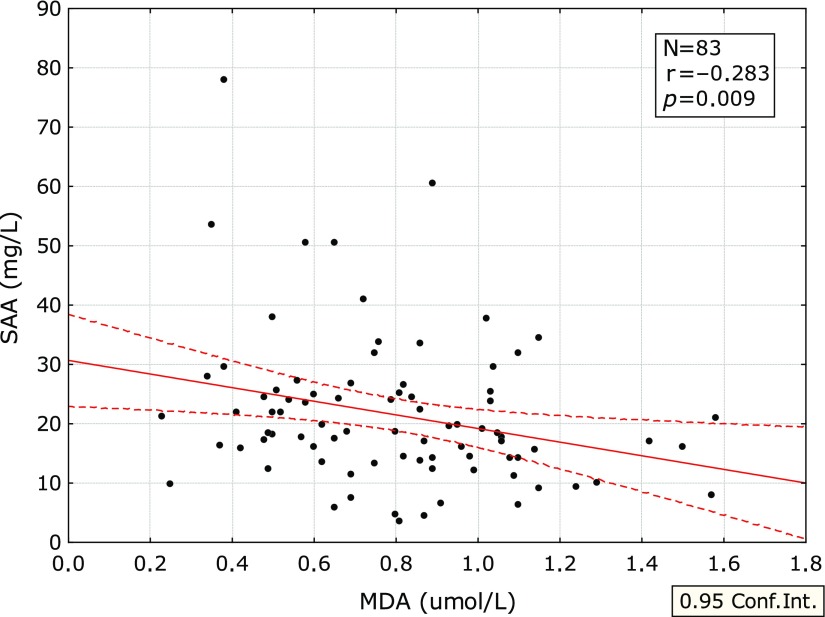
Correlation between MDA and SAA. MDA, malonylaldehyde; SAA, serum amyloid A.

**Fig. 2:**
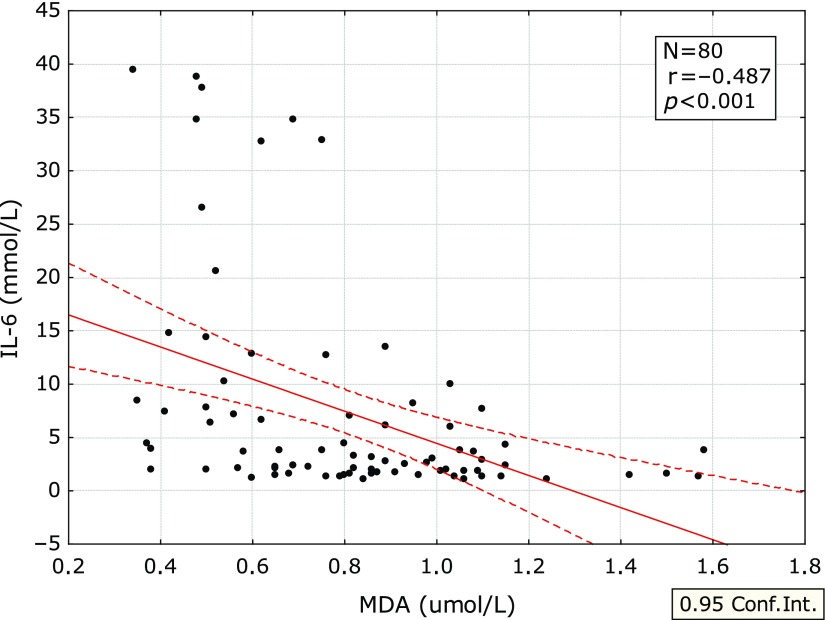
Correlation between MDA and IL-6. IL-6, interleukin 6; MDA, malonylaldehyde.

The correlations of MDA with the inflammatory parameters are summarized in Table 4. The correlation between MDA and SAA is shown on Fig. 1 and between MDA and IL-6 in Fig. 2.

## Discussion

According to the previous reports oxidative stress and parameters of inflammatory activity are increased in the patients suffering from cystic fibrosis.^(5,6)^ Moreover in many subjects malnutrition has the significant impact on the course of this disease.^(7,8)^ It is a reason why nutrition support is indicated and applied frequently by sipping and only in minority of the patients by enteral tube or using parenteral nutrition.

The principal aim of nutritional support is to keep energy and protein balance in order to prevent the progression of malnutrition and the consequent complications. The substitution of pancreatic enzymes is important because the patients with cystic fibrosis have pancreatic insufficiency and therefore the application of oligopeptides preparations may be beneficial.^(8)^ However, the position of immunonutrition has not yet been evaluated in the patients with cystic fibrosis despite the positive impact of arginine^(9–11)^ and omega 3 fatty acids^(12–14)^ on parameters of oxidative stress as well as inflammatory activity was described. The patients with cystic fibrosis are known to have the low plasmatic arginine concentration.^(11)^

Impact is the enteral nutrition preparation with arginine, omega 3 fatty acids and nucleotides. In our study the effect of Impact and Nutridrink on oxidative stress and selected inflammatory parameters was compared. The caloric intake of both preparations given to the patients was identical. After 8 weeks of Impact application the significant drop of systolic and diastolic blood pressure and heart rate was recorded. These changes can be explained by the influence of arginine.^(15)^ When the patients returned to Nutridrink sipping the measured parameters returned back to the previous level.

After eight weeks of Impact application SAA concentration significantly decreased but MDA significantly increased. After the patients returned to Nutridrink, MDA dropped back and the concentration of serum amyloid A went up. SAA was above the upper limit of normal values in all the patients while MDA concentrations remained in normal range even during Impact application. The drop of SAA might have the significant clinical consequences as it was shown that SAA is an indicator of lung infection in cystic fibrosis.^(16)^ Moreover even a brief elevation of SAA is sufficient to accelerate atherosclerosis.^(17)^

The significant negative correlation was found between concentration of MDA and SAA if all the laboratory examinations were evaluated. The significant negative correlations were found also between MDA and some other inflammatory parameters—hsCRP, IL-6, IgA, and IgG. SAA is known to be activated by IL-6.^(18,19)^

In one previous study the increment of MDA concentration was reported after successful treatment of infection exacerbation and the institution of pancreatic enzymes in malnourished patients with cystic fibrosis.^(20)^ However, no such change was recorded in the patients who had substitution of pancreatic enzymes before infection exacerbation. The authors anticipated that due to the substitution of pancreatic enzymes the resorption and utilization of nutritional substrates improved. In chronically malnourished patients it accelerated metabolic turnover and therefore increased oxidative stress. At the same time these patients demonstrated the improving of T lymphocytes function.^(20)^

The explanation of MDA and inflammatory parameters changes in our patients has to be different as the majority of the patients received pancreatic enzymes and no patient with acute infection exacerbation entered the study.

The application of immunonutrients may improve immune response in chronically malnourished and immunocompromised patients and through this way decrease sensitivity to the infection complications. The drop of SAA after Impact could be the result of such positive influence of immunonutrition. However, in malnourished patients the incorporation of immune substrates may be associated with the higher metabolic demand. This could explain the higher MDA concentration. However, it is important to realize that Impact is a mixture of more substrates which may have the different effect on the parameters of the inflammation and the oxidative stress.

It is important to underline there are limitations of our study. Apart from the limited number of the patients, it was only clinical pilot nonrandomized observation. Nevertheless we believe the results could have the clinical implications and should stimulate for the next larger and longer lasted studies that could evaluate the impact of immunonutrition on the risk of infection complications, respiratory function, nutrition status and the patient prognosis suffering from cystic fibrosis.

## Figures and Tables

**Table 1:** Comparison of composition of Nutridrink and Impact—energy intake 100 kcal

	Nutridrink	Impact
Protein	4 g (16%)	5.6 g (22%)
Sacharides	12.2 g (49%)	13.3 g (53%)
Fats	3.9 g (35%)	2.8 g (25%)
Fibre	0.4 g	1.0 g
l-Arginine		1.27 g
Polyunsaturated fatty acids		0.65 g
ω3 polyunsaturated fatty acids		0.32 g

**Table 2:** The changes of antropometric and hemodynamic parameters with relation to nutrition support

	Nutridrink	Impact	Return to Nutridrink	*p*
Body weight (kg)	57 ± 5.8	57 ± 5.9	58 ± 5.8	NS
Systolic BP (mmHg)	115 ± 11	110 ± 7	117 ± 11	0.012
Diastolic BP (mmHg)	69 ± 9	64 ± 8	69 ± 9	0.03
Heart rate (pulse/min)	88 ± 15	77 ± 17	80 ± 14	0.002
BMI (kg/m^2^)	18.8 ± 1.7	18.9 ± 1.6	19 ± 1.6	NS

BP, blood pressure; BMI, body mass index; *p*, statistic significance between Nutridrink and Impact at the beginning of study and after 8 weeks of immunonutrition.

**Table 3:** The changes of the inflammatory parameters with relation to nutrition support

	Nutridrink	Impact	Return to Nutridrink	*p*
MDA (uM)	0.66 ± 0.24	0.96 ± 0.30	0.77 ± 0.27	<0.01
SAA (mg/L)	26.1 ± 16.5	18.1 ± 7.1	21.2 ± 11.9	0.014
CRP (mg/L)	13.7 ± 24.1	17.7 ± 21.1	24.7 ± 42.8	NS
IL-6 (mM)	8.4 ± 11.9	6.1 ± 7.7	7.5 ± 9.2	NS
IL-1 (mM)	10.3 ± 6.6	11.5 ± 8.3	9.1 ± 4.9	NS
IgM (g/L)	1.7 ± 0.7	1.6 ± 0.7	1.8 ± 0.8	NS
IgA (g/L)	3.5 ± 1.8	3.5 ± 1.8	3.7 ± 1.9	NS
IgG (g/L)	16.1 ± 4.1	16.0 ± 4.5	16.6 ± 3.8	NS

MDA, malonyldialdehyd; SAA, serum amyloid A; CRP, C-reactive protein; IL-6, interleukin 6; IL-1, interleukin 1; *p*, statistic significance between Nutridrink and Impact at the beginning of the study and after 8 weeks of immunonutrition.

**Table 4:** Correlation of MDA with the inflammatory parameters

	IgG	IgM	IgA	IL-1	IL-6	SAA	CRP
MDA – cc	–0.574	–0.187	–0.319	–0.041	–0.487	–0.283	–0.436
*p*	<0.001	0.096	0.003	0.717	<0.001	0.009	<0.001

MDA, malonyldialdehyd; SAA, serum amyloid A; CRP, C-reactive protein; IL-6, interleukin 6; IL-1, interleukin 1; cc, correlation coefficient.
